# Peripheral blood mononuclear cells reactivity in recent-onset type I diabetes patients is directed against the leader peptide of preproinsulin, GAD65_271-285_ and GAD65_431-450_

**DOI:** 10.3389/fimmu.2023.1130019

**Published:** 2023-03-09

**Authors:** Rita D. Jores, Davide Baldera, Enrico Schirru, Sandro Muntoni, Rossano Rossino, Maria F. Manchinu, Maria F. Marongiu, Cristian A. Caria, Carlo Ripoli, Maria R. Ricciardi, Francesco Cucca, Mauro Congia

**Affiliations:** ^1^ Department Outpatient Clinic, ASL8 Outpatient Clinic Quartu Sant’Elena, Cagliari, Italy; ^2^ Centro Servizi di Ateneo per gli Stabulari (CeSaSt), University of Cagliari, Monserrato, Italy; ^3^ Department of Biomedical Science, University of Cagliari, Monserrato, Italy; ^4^ Department of Pediatrics, Clinic of Pediatric and Rare Diseases, Microcitemico Pediatric Hospital, A.Cao, ASL8, Cagliari, Italy; ^5^ Department of Medical Science and Public Health, University of Cagliari, Monserrato, Italy; ^6^ Department of Biomedical Sciences, Institute for Genetic and Biomedical Research, Monserrato, Italy; ^7^ Department of Pediatric, Diabetologic Unit, Microcitemico Pediatric Hospital, A.Cao, ASL8, Cagliari, Italy; ^8^ Department of Biomedical Science, University of Sassari, Sassari, Italy

**Keywords:** children, type 1 diabetes mellitus, epitopes, glutamate decarboxylase, preproinsulin, human leukocyte antigen, Sardinia

## Abstract

**Introduction:**

T cell reactivity against pancreatic autoantigens is considered one of the main contributors to the destruction of insulin-producing cells in type 1 diabetes (T1D). Over the years, peptide epitopes derived from these autoantigens have been described in NOD mice and in both HLA class II transgenic mice and humans. However, which ones are involved in the early onset or in the progressive phases of the disease is still unclear.

**Methods:**

In this work we have investigated, in early-onset T1D pediatric patients and HLA-matched controls from Sardinia, the potential of preproinsulin (PPI) and glutamate decarboxylase 65 (GAD65)-derived peptides to induce spontaneous T cell proliferation responses of peripheral blood mononuclear cells (PBMCs).

**Results:**

Significant T cell responses against PPI1-18, PPI7-19 and PPI31-49, the first two belonging to the leader sequence of PPI, and GAD65271-285 and GAD65431-450, were found in HLA-DR4, -DQ8 and -DR3, -DQ2 T1D children.

**Conclusions:**

These data show that cryptic epitopes from the leader sequence of the PPI and GAD65271-285 and GAD65431-450 peptides might be among the critical antigenic epitopes eliciting the primary autoreactive responses in the early phases of the disease. These results may have implications in the design of immunogenic PPI and GAD65 peptides for peptide-based immunotherapy.

## 1 Introduction

Type 1 diabetes mellitus (T1D) is the result of a slow progressive multistep autoimmune destruction of pancreatic insulin-producing cells (1–3). Early studies in European and derived populations have shown that susceptibility to T1D is strongly associated with HLA-DR3, -DQ2 and HLA-DR4, -DQ8 haplotypes, while protection is associated with HLA-DR2, -DQ6 haplotypes (4, 5). Subsequent studies have shown that within these haplotypes certain HLA-DQB1, and -DRB1 alleles and residues confer susceptibility, while others provide resistance to the disease (6–11). For instance, several studies have shown that differences of a few amino acid residues in HLA-DRB1*04 alleles are *per se* sufficient to modify the risk of developing T1D conferred by the high-risk HLA-DQB1*03:02 allele (12–15). Indeed, the spectrum of HLA-DQB1 and -DRB1 association, which constitutes the major component of T1D risk, is more complex than initially outlined and can be primarily grouped into very high risk, intermediate and very low risk haplotypes which, in turn, are the result of the structure of the peptide-binding pockets (15, 16).

In recent years, the intricacy and the molecular nature of immunogenic T cell epitopes of pancreatic autoantigens have been further elucidated (17). It is generally believed that T cell responses against autoantigens may become less evident with the progressive destruction of the islets of Langerhans (18, 19). Moreover, the spreading of T cell reactivity to other not primarily involved regions, of preproinsulin (PPI) or glutamate decarboxylase 65 (GAD65) autoantigens or to other pancreatic proteins/neoantigens generated from post-translational modifications may be an important confounding factor complicating the identification of pathogenically relevant T cells (19–22). Secondly, autoreactive immune responses are not completely disease-specific, since T cell reactivity against autoantigenic proteins is detected also in control subjects who carry disease-associated HLA-DR and -DQ molecules (18, 23, 24).

A useful tool to identify immunodominant T cell epitopes from pancreatic autoantigens is the use of humanized HLA transgenic mice (25). Indeed, numerous PPI and GAD65 T cell epitopes have been identified in triple HLA, human CD4 (hCD4) and IA knock-out transgenic mice (25–29) and confirmed in T1D patients (30–32). The majority of these studies have been conducted using triple HLA-DRB1*04:01, hCD4, IA knock-out mice (25–29), in HLA-DQ8 transgenic mice (33), whilst literature regarding studies on patients carrying different HLA-DR4 subtypes, such as the HLA-DRB1*04:05-DQA1*03:01-DQB1*03:02 haplotype is lacking. Interestingly, this is the most frequent HLA-DR4 haplotype found in Sardinian T1D patients (13, 16, 34).

Thus, we tested in children with recent-onset T1D and HLA-matched healthy controls from Sardinia a set of PPI and GAD65 peptides derived from previous studies in both HLA-DR4 and -DQ8 transgenic mice and humans (26–28, 33, 35).

## 2 Materials and methods

### 2.1 Sample selection

Twelve HLA-DR4, -DQ8 positive and four HLA-DR4, -DQ8 negative T1D patients with recent onset disease have been studied. In all T1D patients the diagnosis was performed 3 days before enrollment. Fourteen HLA-DR4, -DQ8 positive and four HLA-DR4, -DQ8 negative healthy blood donors without history of autoimmune diseases served as controls. The 18 healthy individuals were HLA typed in connection with other studies (36). All T1D patients with recent-onset disease were recruited from our clinic in the Microcitemico Hospital A. Cao, ASL8, Cagliari. The mean age was 7.4 ± 3.3 years, in line with the mean age of T1D onset in Sardinia (37). The mean age of healthy controls was 43.6 ± 5.4. **Table 1** shows the HLA-DR, -DQ typing of T1D patients and controls. An ethics committee approval was obtained for this study (authorization no. PG/2016/7815).

**Table 1:** HLA typing, in 16 recent-onset T1D patients and 18 ethnically matched controls.

T1D patients								Healthy controls								
ID	DRB1	DQA1	DQB1	DRB1	DQA1	DQB1		ID	DRB1	DQA1	DQB1	DRB1	DQA1	DQB1
01	*0405	*0301	*0302	*0405	*0301	*0302		01	*0405	*0301	*0302	*1601	*0102	*0502
02	*0405	*0301	*0302					02	*0405	*0301	*0302			
03	*0405	*0301	*0302					03	*0402	*0301	*0302			
04	*0402	*0301	*0302	*0301	*0501	*0201		04	*0405	*0301	*0302			
05	*0405	*0301	*0302	*0301	*0501	*0201		05	*0405	*0301	*0302	*0301	*0501	*0201
06	*0405	*0301	*0302	*0405	*0301	*0302		06	*0402	*0301	*0302			
07	*0405	*0301	*0302	*0101	*0101	*0501		07	*0405	*0301	*0302			
08	*0405	*0301	*0302					08	*0403	*0301	*0302	*0301	*0501	*0201
09	*0405	*0301	*0302	*0301	*0501	*0201		09	*0405	*0301	*0201			
10	*0405	*0301	*0302	*0405	*0301	*0302		10	*0405	*0301	*0201			
11	*0405	*0301	*0302	*0301	*0501	*0201		11	*0404	*0301	*0302	*1101	*0501	*0301
12	*0402	*0301	*0302	*0301	*0501	*0201		12	*0405	*0301	*0302			
13	*0301	*0501	*0201	*1601	*0102	*0502		13	*0405	*0301	*0302			
14	*0301	*0501	*0201	*1601	*0102	*0502		14	*0405	*0301	*0302			
15	*0301	*0501	*0201	*1601	*0102	*0502		15	*0301	*0501	*0201	*0301	*0501	*0201
16	*0301	*0501	*0201	*0301	*0501	*0201		16	*0301	*0501	*0201	*1601	*0102	*0502
								17	*0301	*0501	*0201	*0301	*0501	*0201
								18	*0301	*0501	*0201	*0301	*0501	*0201

### 2.2 HLA class II typing and haplotype analysis

HLA-DRB1, -DQA1 and -DQB1 genotypes were determined by polymerase chain reaction with sequence-specific primers (SSP-PCR) using Olerup SSP typing kits (Olerup SSP AB, Stockholm, Sweden). The HLA class II haplotypes were predicted on the basis of the known linkage disequilibrium in Sardinians (12, 13).

### 2.3 Proliferation assays

Blood was drawn from recent-onset T1D patients within two to three days after diagnosis. Peripheral blood mononuclear cells (PBMCs) were isolated by density gradient. Blood was diluted 1:1 with complete medium (RPMI 1640, Life Technologies Italia, Monza, Italy) containing 2% heat-inactivated fetal calf serum, 2 mM L-glutamine, 100 U/ml penicillin, 100 μg/ml of streptomycin, 50 μM 2-mercaptoethanol and HEPES. Diluted blood was then layered 1:1 on a Lymphoprep™ gradient (Stem Cell Technologies, Monza, Italy) and centrifuged at 400 g for 30 min at room temperature.

Cells were harvested and washed in complete medium containing 10-15% autologous human serum, and 2 x 10^5^ cells/200 µl per well were incubated in the presence or absence of 20 µg/ml of PPI or GAD65 peptides. Proliferative responses against antigens were determined in 6 replicate cultures in round-bottomed 96 well plates by [^3^H]-thymidine incorporation after 6 days of culture, following a 6-hour pulse with 0.5 µCi [^3^H]-thymidine. T cell reactivity was attributed using a relative ratio (RR) of counts per minute (cpm) of radioactivity incorporated by PBMCs plus peptide, compared to PBMCs alone. A RR of 3 or higher was considered positive and used in the statistical calculation. T cell reactivity has been controlled by stimulation with concanavalin A (data not shown).

### 2.4 PPI and GAD65 peptides

Eleven peptides from PPI and 16 from GAD65 (**Table 2**) were purchased from Life Technologies Italia (Monza, Italy). These peptide epitopes were derived from research in humans and from previous studies in HLA-DR4 or -DQ8 transgenic mice (21, 25–33, 35). The purity of all these peptides was verified by reverse-phase HPLC and mass spectroscopic analysis. Before use, peptides were suspended in sterile PBS at a concentration of 2 mg/ml and stored at -80° C.

**Table 2:** PPI and GAD65 immunodominant epitopes identified in different HLA transgenic mice and humans used in this work.

Peptides	Sequences	References
PPI_1-18_	MALWMRLLPLLALLALWG	(27, 33)
PPI_7-19_	LLPLLALLALWGP	(33)
PPI_11-26_	LALLALWGPDPAAAFV	(27, 33)
PPI_13-25_	LLALWGPDPAAAF	(27, 33)
PPI_19-31_	PDPAAAFVNQHLC	(27)
PPI_31-49_	CGSHLVEALYLVCGERGFF	(32)
PPI_40-59_	YLVCGERGFFYTPKTRREAE	(32)
PPI_52-67_	PKTRREAEDLQVGQVE	(35)
PPI_58-70_	AEDLQVGQVELGG	(31, 35)
PPI_73-90_	GAGSLQPLALEGSLQKRG	(27, 32)
PPI_85-101_	SLQKRGIVEQCCTSICS	(27)
GAD65_76-90_	DQKPCSCSKVDVNYA	(35)
GAD65_81-95_	SCSKVDVNYAFLHAT	(30, 35)
GAD65_101-115_	CDGERPTLAFLQDVM	(28, 35)
GAD65_116-130_	MNILLQYVVKSFDRST	(26, 35)
GAD65_206-220_	TYEIAPVFVLLEYVT	(28)
GAD65_271-285_	PRLIAFTSEHSHFSL	(26, 35)
GAD65_356-370_	KYKIWMHVDAAWGGG	(26, 35)
GAD65_376–390_	KHKWKLSGVERANSV	(26, 35)
GAD65_431-450_	KHYDLSYDTGDKALQ	(28)
GAD65_481-495_	LYNIIKNREGYEMVF	(26, 35)
GAD65_511-525_	PSLRTLEDNEERMSR	(26, 35)
GAD65_526-540_	LSKVAPVIKARMMEY	(30)
GAD65_536-550_	RMMEYGTTMVSYQPL	(28, 35)
GAD65_546-560_	SYQPLGDKVNFFRMV	(26, 35)
GAD65_551-565_	GDKVNFFRMVISNPA	(26, 35)
GAD65_556-570_	FFRMVISNPAATHQD	(26, 35)

### 2.5 Statistical analysis

A Chi-square test was used to compare PPI and GAD65 proliferative responses between T1D and controls. In the case of samples size lower than 5, a two-tailed Fisher’s exact test was used. A p value lower than 0.05 was considered statistically significant.

## 3 Results

### 3.1 T cell reactivity against PPI peptides

PBMCs from patients and HLA-matched healthy individuals were cultured with or without peptides and their proliferations measured. The eleven peptides tested cover the PPI protein sequence almost completely. Data were analyzed as a whole and after grouping patients and control individuals according to HLA-DR4 haplotype. **Tables S1**, **S2** summarize the proliferative responses and RR obtained in T1D patients and controls respectively.

We have found that 75% of T1D patients and 33.3% of controls did respond to PPI peptides (p= 0.0204). Response to more than one PPI peptide was found in 62.5% of T1D patients and in 11.1% of controls (p= 0.0033).

**Figure 1:**
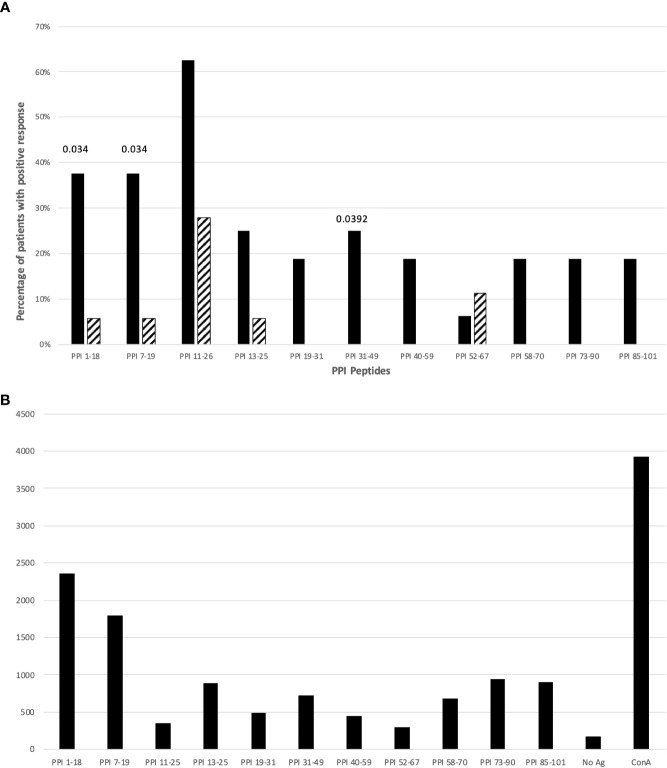
**(A)** Percentage of T1D patients (solid bars) and controls (hatched bars) responding to PPI peptides. Significant p values (<0.05) are indicated above the corresponding bars. Statistical analysis was performed using the Fisher’s exact test. **(B)** The figure shows cpm response with different PPI peptides in a T1D patient (n. 11, **Table S1**).

Next, we evaluated which epitopes accounted for this reactivity by comparing the frequency of responses against specific peptides of PPI in T1D patients and controls. **Figure 1A** illustrates the frequency of responses against PPI peptides, while **Figure 1B** provides an example of responses against PPI peptides in a T1D patient (n. 11, **Table S1**). **Tables S1**, **S2** summarize the proliferative responses and the RR obtained in T1D patients and controls respectively. Significant differences between patients and controls were found for PPI_1-18_ (p=0.0348; **Figure 1A**), PPI_7-19_ peptides (p=0.0348; **Figure 1A**) and PPI_31-49_ (p=0.0392; **Figure 1A**). The statistical significance for PPI peptides was lost after stratification for DR4 haplotypes.

In terms of global reactivity, our results indicate a higher response against peptides of the PPI protein and less reactivity against GAD65 peptides. In T1D patients, we observed 46 out of 176 responses for PPI peptides versus 40 out of 224 responses for GAD65 (p=0.045).

### 3.2 T cell reactivity against GAD65 peptides

Sufficient PBMCs for measurement of proliferative responses against GAD65 peptides were available from 14 T1D patients and 18 control individuals. **Tables S3**, **S4** summarize the proliferative responses and the RR obtained in T1D patients and controls respectively.

**Figure 2:**
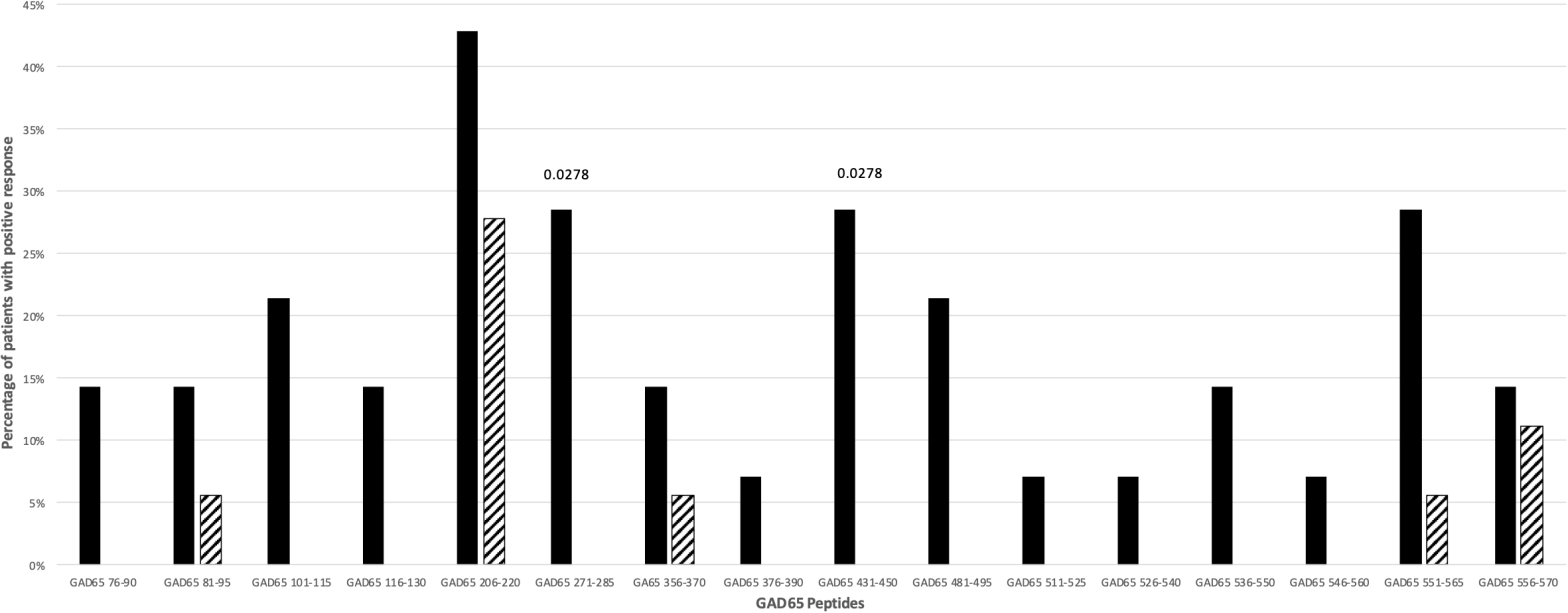
Percentage of T1D patients (solid bars) and controls (hatched bars) responding to GAD65 peptides. Significant p values (<0.05) are indicated above the corresponding bars. Statistical analysis was performed using the Fisher’s exact test.

Fifty-six percent of T1D patients and 27.7% of controls did respond against GAD65 peptides (p: NS). The responses to more than one GAD65 peptide did not differ significantly between T1D patients (37.5%) and controls (16.6%). Only for GAD65_271-285_ and GAD65_431-450_ peptides a significant difference between patients and controls was observed (p=0.0278; **Figure 2**). The statistical significance for these two GAD65 peptides was lost after stratification for DR4 haplotypes.

## 4 Discussion

Here, we report the capacity of PPI and GAD65 derived peptides obtained from humans and from HLA-DR4 and -DQ8 transgenic mice (21, 25–33, 35) to induce spontaneous T cell proliferation responses in PBMCs from recent-onset T1D pediatric patients.

In terms of global reactivity, our results indicate a higher response against peptides of the PPI protein and less reactivity against GAD65 peptides.

Analysis of proliferative responses to PPI showed that reactivity is mainly directed against PPI_1-18_, and PPI_7-19_ peptides of the leader sequence. These 2 peptides are included in the immunodominant peptide PPI_1-24_ described as HLA-DQ8-restricted in HLA-DQ8 transgenic mice (33). However, we found responses against these 2 peptides in both HLA-DR4, -DQ8 and -DR3, -DQ2 positive T1D patients compared to HLA-matched controls (**Tables S1, S2**; **Figure 1A**).

Since we found significant responses for both peptides, we hypothesize that the sequence responsible for reactivity in T1D patients is included in the PPI_7-19_ shared sequence LLPLLALLALWG. Prevalent reactivity of T1D PBMCs against the leader sequence may indicate that precursors of insulin, normally confined to the ER of the β-cells, are involved in targeting T cell autoreactivity to the islets. Interestingly, the same region of PPI is recognized by CD8+ T cells from recent-onset T1D patients (38, 39), which may suggest that the leader sequence of PPI is targeted by both CD4+ and CD8+ autoreactive T cells. In addition to HLA-DQ8, the leader sequence of PPI (LALLALWGPDPAAAFV) may be presented also by HLA-DRB1*04:01 as previously shown in HLA-DRB1*04:01 transgenic mice (27). Therefore, in analogy to other autoantigens such as GAD65 (26, 28), the leader sequence of PPI may show overlapping peptide sequences that are presented by both HLA-DR and -DQ molecules.

Also for PPI_31–49_ peptide, including the B chain peptide PPI_B9-23_, reported immunodominant in both humans and NOD mice (40–42), significant proliferative responses were observed (**Table S1**; **Figure 1A**).

Interestingly, CD8+ T cells in long-standing patients have been shown to recognize the B-chain peptide PPI_33–42_ (38), that is also targeted by both human and NOD mice CD4+ T cells (41, 42).

Among the 16 GAD65 peptides tested, only GAD65_271-285_ and GAD65_431-450_ peptides induced significant proliferative responses in T1D patients. These patients carried both HLA-DR4 and HLA-DR3 haplotypes (**Tables S3, S4**; **Figure 2**). These findings are in line with previous data showing that GAD65_271-285_ was found immunodominant in HLA-DRB1*04:01 transgenic mice and humans (26), whilst a peptide similar to GAD65_431-450_ (GAD65_431-445_) is recognized by HLA-DQ8 molecules in HLA-DQ8 transgenic mice (28). Interestingly, GAD65_431-450_ has been also reported as an inducer of IL-13 in CD4+ T cell lines derived from HLA-DR3, -DQ2 homozygous individuals (43).

Noteworthy, the GAD65 region 245-450 that includes both GAD65_271-285_ and GAD65_431-450_ has been identified as the main target of the earlier anti-GAD65 response in pre-diabetic, healthy high-risk subjects and early onset T1D patients (44–46). Finally, in genetically at-risk T1D patients, autoantibodies against GAD65 are found mainly in HLA-DR3, -DQ2 and less commonly in HLA-DR4, -DQ8 patients (19, 47).

Our findings demonstrate the crucial importance of validating in T1D patients the prediction of immunogenic epitopes. Indeed, even epitopes that are immunodominant in HLA class II transgenic mice may be not immunogenic in humans. As an example, GAD65_206-220_ peptide is an immunogenic/immunodominant T cell epitope in HLA-DQ8 transgenic mice (28, 48) and in NOD mice after immunization with murine GAD65 (49) and, as such, could be considered a good pathogenic candidate for T1D in mice and humans. Instead, T cell receptor transgenic mice specific for GAD65_206-220_ have been demonstrated to be protected against T1D development (50). In accordance, our data show that this peptide elicits proliferative responses not only in T1D patients but also in controls (**Tables S3, S4**; **Figure 2**), making a pathogenic role highly unlikely also in humans.

Our study has several limitations that we tried to mitigate. The large number of potential T cell epitopes of pancreatic autoantigenic proteins that need to be tested challenges the relatively small amount of blood that can be drawn from children. To circumvent this limitation, we restricted the number of epitopes by prior analysis of the T cell responses in transgenic mice carrying human HLA haplotypes. Another limitation is the T cell spreading in long standing T1D patients (19–21), which is why we focused on early onset T1D pediatric patients recruited within three to four days after diagnosis. Discrepancies in the interpretation of T cell responses from PBMCs, the usage of diverse experimental methodologies among laboratories and the variability in the frequencies of HLA-DR4 subtypes in different T1D populations, may lead to different results. In this work we tried to reduce these variables taking advantage of the genetically homogenous Sardinian population, less prone to both genetic and clinical confounds that are present in more cosmopolitan collections (13, 51). Finally, a further possible limitation of this work was the significant age difference between T1D patients and controls. However, the 18 controls were healthy blood donors who were selected for a negative history of autoimmune diseases, thus making spontaneous not specific responses less likely than what could have been derived from age matched random pediatric controls attending the outpatient clinic for different disorders.

In summary, we identified for both PPI and GAD65 specific proliferative responses addressed to the PPI leader sequence and to GAD65_271-285_, GAD65_431-450_ in T1D patients. We suggest that these could be primary epitopes involved in the early phase of the clinical onset of T1D. Finally, these data hint towards the possibility that the leader sequence of PPI is targeted by both CD8+ and CD4+ T cells, with HLA class I and class II presenting slightly different overlapping peptide epitopes and thus inducing a stronger pathogenic immune response involved in the β-cell destruction.

These results may have implications in the design of immunogenic PPI and GAD65 peptides for peptide-based immunotherapy. Finally, this work could pave the way to test these epitopes in early stage T1D patients (stage 1 or 2) who have islet autoantibodies but no overt clinical diabetes requiring treatment with exogenous insulin.

## Data availability statement

The original contributions presented in the study are included in the article/**Supplementary Material**. Further inquiries can be directed to the corresponding author.

## Ethics statement

The studies involving human participants were reviewed and approved by Comitato Etico Indipendente AOUCA. Written informed consent to participate in this study was provided by the participants’ legal guardian/next of kin.

## Author contributions

RJ and MC conceptualized, designed and led the research. DB, ES, SM contributed to data interpretation and drafting the manuscript. RR provided HLA typing. MMan, MMar and CC collected and processed PBMC. CR and MR provided T1D patients. FC critically revised the manuscript. MC is the guarantor of this study. All authors contributed to the article and approved the submitted version.
