# Supplementary material for: Peripheral blood mononuclear cells reactivity in recent-onset type I diabetes patients is directed against the leader peptide of preproinsulin, GAD65271-285 and GAD65431-450
Source: Front Immunol. 2023 Mar 9;14:1130019. doi: 10.3389/fimmu.2023.1130019 (PMC10034372; doi:10.3389/fimmu.2023.1130019)
Supplement: Supplementary file 1 [file Table_1.docx]

|  | | HLA-DR4/DR4 or HLA-DR4/X | | | | | | | | | | | | HLA-DR3/DR3 or HLA-DR3/X | | | |
| --- | --- | --- | --- | --- | --- | --- | --- | --- | --- | --- | --- | --- | --- | --- | --- | --- | --- |
| ID 🡪 | | 01 | 02 | 03 | 04 | 05 | 06 | 07 | 08 | 09 | 10 | 11 | 12 | 13 | 14 | 15 | 16 |
| PPI 1-18 | cpm | 1644.2 | 1775.3 | 423.7 | 2119.5 | 443.4 | 4767.5 | 1090 | 1044.8 | 2642.5 | 3739.3 | 2363 | 990 | 338.7 | 1680.5 | 606.5 | 6006.8 |
|  | RR | 2.7 | 1.2 | 2.5 | 3.5 | 1.3 | 2.3 | 0.8 | 1.4 | 2.6 | 5.8 | 14.0 | 5.9 | 1.7 | 4.1 | 5.0 | 1.5 |
| PPI 7-19 | cpm | 2331.6 | 1900.3 | 303.8 | 1663.2 | 362.8 | 6654 | 1387.4 | 747.2 | 996.2 | 3189.3 | 1796.3 | 749.5 | 159.2 | 1618.8 | 313.2 | 3585.8 |
|  | RR | 3.8 | 1.3 | 1.8 | 2.8 | 1.0 | 3.2 | 1.0 | 1.0 | 1.0 | 5.0 | 10.6 | 4.5 | 0.8 | 3.9 | 2.6 | 0.9 |
| PPI 11-26 | cpm | 3059.2 | 6381.2 | 3905.4 | 4906.6 | 712.6 | 7752.3 | 1006.6 | 8362.8 | 2028 | 1752.5 | 357 | 972.5 | 1318.3 | 2846.8 | 668.8 | 7735.2 |
|  | RR | 5.0 | 4.4 | 23.2 | 8.2 | 2.1 | 3.7 | 0.7 | 11.1 | 2.0 | 2.7 | 2.1 | 5.8 | 6.6 | 6.9 | 5.5 | 1.9 |
| PPI 13-25 | cpm | 658.2 | 2445.3 | 574.5 | 1742.9 | 191.6 | 3716.8 | 2841.4 | 2282.8 | 1492.2 | 733.2 | 896.2 | 537.7 | 205 | 774 | 209.4 | 5141.8 |
|  | RR | 1.1 | 1.7 | 3.4 | 2.9 | 0.6 | 1.8 | 2.0 | 3.0 | 1.5 | 1.1 | 5.3 | 3.2 | 1.0 | 1.9 | 1.7 | 1.3 |
| PPI 19-31 | cpm | 661.2 | 1276.3 | 445 | 2084.7 | 248 | 2394.7 | 1232.2 | 783.5 | 1175.7 | 2902.8 | 488.8 | 666.2 | 199.8 | 393.7 | 241.2 | 5948.2 |
|  | RR | 1.1 | 0.9 | 2.6 | 3.5 | 0.7 | 1.2 | 0.9 | 1.0 | 1.1 | 4.5 | 2.9 | 4.0 | 1.0 | 1.0 | 2.0 | 1.5 |
| PPI 31-49 | cpm | 631.2 | 1685.2 | 246.2 | 2219.8 | 313.6 | 1390.3 | 441.6 | 1511.8 | 853.7 | 1834 | 721.3 | 586.2 | 216.8 | 1359.3 | 232.3 | 4764.2 |
|  | RR | 1.0 | 1.2 | 1.5 | 3.7 | 0.9 | 0.7 | 0.3 | 2.0 | 0.8 | 2.9 | 4.3 | 3.5 | 1.1 | 3.3 | 1.9 | 1.2 |
| PPI 40-59 | cpm | 800.2 | 1657.3 | 782.8 | 1758.9 | 225.2 | 3912.7 | 2725 | 1072.8 | 803.5 | 4518.5 | 448.3 | 260.2 | 127.8 | 1378.8 | 176.8 | 3766.2 |
|  | RR | 1.3 | 1.1 | 4.7 | 2.9 | 0.7 | 1.9 | 1.9 | 1.4 | 0.8 | 7.1 | 2.6 | 1.6 | 0.6 | 3.3 | 1.5 | 0.9 |
| PPI 52-67 | cpm | 421.5 | 1786.2 | 332.2 | 1403.9 | 197.5 | 2538.8 | 2214.2 | 667.8 | 719.8 | 1735.2 | 303.7 | 258.7 | 128.3 | 1705.8 | 105.2 | 5890 |
|  | RR | 0.7 | 1.2 | 2.0 | 2.3 | 0.6 | 1.2 | 1.5 | 0.9 | 0.7 | 2.7 | 1.8 | 1.5 | 0.6 | 4.1 | 0.9 | 1.5 |
| PPI 58-70 | cpm | 1373.8 | 1848 | 440.5 | 1722.9 | 209.8 | 3076.5 | 1444.4 | 1138.3 | 769.5 | 3845 | 679 | 845.5 | 218.5 | 714 | 183.3 | 5358 |
|  | RR | 2.3 | 1.3 | 2.6 | 2.87 | 0.6 | 1.5 | 1.0 | 1.5 | 0.8 | 6.0 | 4.0 | 5.0 | 1.1 | 1.7 | 1.5 | 1.3 |
| PPI 73-90 | cpm | 751.5 | 2139.2 | 414.2 | 1724.7 | 213.6 | 1571.3 | 1530.8 | 1078.2 | 922.5 | 4152.5 | 945 | 547.3 | 192.7 | 657.2 | 94.8 | 6325.2 |
|  | RR | 1.2 | 1.5 | 2.5 | 2.9 | 0.6 | 0.8 | 1.1 | 1.4 | 0.9 | 6.5 | 5.6 | 3.3 | 1.0 | 1.6 | 0.8 | 1.6 |
| PPI 85-101 | cpm | 941 | 1490.7 | 487.8 | 1301.2 | 258 | 2257.7 | 2362.2 | 701.5 | 726.7 | 3376.8 | 906.3 | 1034 | 173.8 | 557.7 | 187.5 | 4604 |
|  | RR | 1.5 | 1.0 | 2.9 | 2.2 | 0.7 | 1.1 | 1.6 | 0.9 | 0.7 | 5.3 | 5.4 | 6.2 | 0.9 | 1.3 | 1.6 | 1.1 |
| No Ag | cpm | 607.2 | 1448 | 168.2 | 600.1 | 345.6 | 2077.1 | 1434.4 | 754.5 | 1025.1 | 639.3 | 169.3 | 167.7 | 200.4 | 413.3 | 120.7 | 4053.2 |

**Supplementary Table 1.** Proliferative responses (cpm) and relative ratios (RR) against PPI peptides compared to absence of peptides stimulation (No Ag) in 16 T1D patients. The thick vertical line separates 12 DR4 positive from 4 DR4 negative patients. Patients are identified using a numerical ID; below each ID, the cpm and RR are shown. All values are derived from sextuplet cultures. Shaded areas represent responses that were considered positive (RR≥3) and used in the statistical analysis.

|  | | HLA-DR4/DR4 or HLA-DR4/X | | | | | | | | | | | | | | HLA-DR3/DR3 or HLA-DR3/X | | | |
| --- | --- | --- | --- | --- | --- | --- | --- | --- | --- | --- | --- | --- | --- | --- | --- | --- | --- | --- | --- |
| ID 🡪 | | 01 | 02 | 03 | 04 | 05 | 06 | 07 | 08 | 09 | 10 | 11 | 12 | 13 | 14 | 15 | 16 | 17 | 18 |
| PPI 1-18 | cpm | 354.5 | 4340.3 | 3004.8 | 1939.3 | 1685.8 | 3042.2 | 4191 | 2021.7 | 1799 | 1749.7 | 439.3 | 631.8 | 1294 | 1939.5 | 488.5 | 495.3 | 777.2 | 1827.8 |
|  | RR | 0.3 | 0.9 | 0.8 | 0.5 | 3.4 | 1.7 | 1.4 | 1.9 | 0.5 | 0.3 | 2.3 | 1.5 | 1.6 | 0.8 | 0.5 | 1.7 | 2.0 | 1.5 |
| PPI 7-19 | cpm | 1513.2 | 5718.3 | 2124.8 | 2349.2 | 1008.2 | 3308.7 | 3370.3 | 3249.8 | 3990.3 | 3957.7 | 550.5 | 464.2 | 636 | 1856 | 835 | 532.3 | 1018.5 | 1376 |
|  | RR | 1.2 | 1.2 | 0.5 | 0.6 | 2.0 | 1.8 | 1.1 | 3.1 | 1.0 | 0.8 | 2.9 | 1.1 | 0.8 | 0.8 | 0.8 | 1.8 | 2.6 | 1.1 |
| PPI 11-26 | cpm | 3709.8 | 5584.5 | 9405.5 | 2070.2 | 3221.5 | 12220.2 | 8016.3 | 7739.8 | 916.2 | 509.5 | 616.5 | 1031.2 | 2605.3 | 1710 | 756.3 | 544.5 | 1044.7 | 3149.2 |
|  | RR | 2.9 | 1.1 | 2.4 | 0.5 | 6.5 | 6.7 | 2.6 | 7.3 | 0.2 | 0.1 | 3.3 | 2.5 | 3.1 | 0.7 | 0.8 | 1.9 | 2.7 | 2.6 |
| PPI 13-25 | cpm | 551 | 8152.2 | 5474.8 | 2943 | 1587.5 | 4037.2 | 5852.3 | 2675 | 5452.8 | 6035.5 | 266.8 | 774 | 583.8 | 2271.7 | 1091.2 | 756.3 | 446.7 | 1164.3 |
|  | RR | 0.4 | 1.7 | 1.4 | 0.7 | 3.2 | 2.2 | 1.9 | 2.5 | 1.4 | 1.2 | 1.4 | 1.9 | 0.7 | 0.9 | 1.1 | 2.6 | 1.1 | 1.0 |
| PPI 19-31 | cpm | 1485.8 | 6614.8 | 3618 | 4372.8 | 890.7 | 3463 | 6146.7 | 1272.7 | 2939.8 | 4193.7 | 149.2 | 598.3 | 1005.3 | 3168 | 884.2 | 387 | 502.2 | 883.2 |
|  | RR | 1.2 | 1.3 | 0.9 | 1.0 | 1.8 | 1.9 | 2.0 | 1.2 | 0.8 | 0.8 | 0.8 | 1.4 | 1.2 | 1.3 | 0.9 | 1.3 | 1.3 | 0.7 |
| PPI 31-49 | cpm | 1093.2 | 7735 | 3471.8 | 3228.7 | 669.3 | 2751.5 | 3661.7 | 1016.5 | 4937.8 | 8126.2 | 331.2 | 721 | 718.5 | 2870.8 | 1001.7 | 325 | 436.3 | 1814.7 |
|  | RR | 0.9 | 1.6 | 0.9 | 0.8 | 1.3 | 1.5 | 1.2 | 1.0 | 1.3 | 1.6 | 1.8 | 1.7 | 0.9 | 1.2 | 1.0 | 1.1 | 1.1 | 1.5 |
| PPI 40-59 | cpm | 1136 | 4009.8 | 3221.5 | 3702 | 983.8 | 2566.3 | 8073.5 | 743.5 | 1603 | 5043.3 | 271.5 | 524.8 | 835.5 | 3190.7 | 864.7 | 519.3 | 536 | 935.2 |
|  | RR | 0.9 | 0.8 | 0.8 | 0.9 | 2.0 | 1.4 | 2.6 | 0.7 | 0.4 | 1.0 | 1.4 | 1.3 | 1.0 | 1.3 | 0.9 | 1.8 | 1.4 | 0.8 |
| PPI 52-67 | cpm | 882.5 | 7256.8 | 3205 | 5082.3 | 386.7 | 1051.5 | 5145.5 | 3523.2 | 2262 | 5879.2 | 476.7 | 586.7 | 322 | 3796.3 | 1744 | 1057.7 | 319.5 | 939 |
|  | RR | 0.7 | 1.5 | 0.8 | 1.2 | 0.8 | 0.6 | 1.7 | 3.3 | 0.6 | 1.2 | 2.5 | 1.4 | 0.4 | 1.6 | 1.7 | 3.7 | 0.8 | 0.8 |
| PPI 58-70 | cpm | 956.3 | 3966.5 | 2618 | 3659.5 | 1044.8 | 2458.3 | 5515.3 | 2165.5 | 806.4 | 4932.3 | 196 | 575 | 1074.5 | 1844.3 | 1211 | 225.7 | 305.7 | 720.8 |
|  | RR | 0.7 | 0.8 | 0.7 | 0.9 | 2.1 | 1.3 | 1.8 | 2.1 | 0.2 | 1.0 | 1.0 | 1.4 | 1.3 | 0.8 | 1.2 | 0.8 | 0.8 | 0.6 |
| PPI 73-90 | cpm | 1396.8 | 13032 | 2980.2 | 5079 | 638.3 | 1584 | 3874.2 | 1015.2 | 3179.7 | 6006.8 | 560.2 | 534.8 | 435.2 | 3324.8 | 1554.2 | 259.3 | 380.2 | 481.2 |
|  | RR | 1.1 | 2.7 | 0.8 | 1.2 | 1.3 | 0.9 | 1.3 | 1.0 | 0.8 | 1.2 | 3.0 | 1.3 | 0.5 | 1.4 | 1.5 | 0.9 | 1.0 | 0.4 |
| PPI 85-101 | cpm | 1180.3 | 6689.7 | 2555.3 | 2615.3 | 692.8 | 3010 | 3864.2 | 993.5 | 1394.8 | 6563.2 | 476 | 429.7 | 743.7 | 1696.7 | 1175.7 | 255.8 | 443.8 | 1121.7 |
|  | RR | 0.9 | 1.4 | 0.6 | 0.6 | 1.4 | 1.6 | 1.3 | 0.9 | 0.4 | 1.3 | 2.5 | 1.0 | 0.9 | 0.7 | 1.2 | 0.9 | 1.1 | 0.9 |
| No Ag | cpm | 1278.5 | 4909.6 | 3952.2 | 4222.2 | 496.8 | 1836.7 | 3086.3 | 1053.4 | 3817.8 | 5051.5 | 187.8 | 415.8 | 834.2 | 2423.8 | 1007.8 | 288.8 | 393.1 | 1199.5 |

**Supplementary Table 2.** Proliferative responses (cpm) and relative ratios (RR) against PPI peptides compared to absence of peptides stimulation (No Ag) in 18 healthy controls. The thick vertical line separates 14 DR4 positive from 4 DR4 negative individuals. Control individuals are identified using a numerical ID; below each ID, the cpm and RR are shown. All values are derived from sextuplet cultures. Shaded areas represent responses that were considered positive (RR≥3) and used in the statistical analysis.

|  | | HLA-DR4/DR4 or HLA-DR4/X | | | | | | | | | | | HLA-DR3/DR3 or HLA-DR3/X | | |
| --- | --- | --- | --- | --- | --- | --- | --- | --- | --- | --- | --- | --- | --- | --- | --- |
| ID 🡪 | | 02 | 03 | 04 | 05 | 06 | 07 | 08 | 09 | 10 | 11 | 12 | 13 | 14 | 16 |
| GAD 76-90 | cpm | 1300.2 | 421.2 | 3261.2 | 656.4 | 2398.2 | 3129.6 | 382.2 | 754.2 | 1553.7 | 2039.2 | 312 | 145.5 | 638.8 | 141.6 |
|  | RR | 0.9 | 2.5 | 5.4 | 1.9 | 1.2 | 2.2 | 0.5 | 0.7 | 2.4 | 12.0 | 1.9 | 0.7 | 1.5 | 0.0 |
| GAD 81-95 | cpm | 790.7 | 349.2 | 1860.8 | 429.4 | 585.7 | 2446.6 | 1234.2 | 1543.5 | 889.5 | 1853.5 | 145.2 | 170.3 | 428.7 | 251.2 |
|  | RR | 0.5 | 2.1 | 3.1 | 1.2 | 0.3 | 1.7 | 1.6 | 1.5 | 1.4 | 10.9 | 0.9 | 0.8 | 1.0 | 0.1 |
| GAD 101-115 | cpm | 797.5 | 899.3 | 1891.2 | 496.4 | 1817.7 | 3983.6 | 1632.8 | 1005.8 | 4374.8 | 413.8 | 396.2 | 151.4 | 787 | 211 |
|  | RR | 0.6 | 5.3 | 3.2 | 1.4 | 0.9 | 2.8 | 2.2 | 1.0 | 6.8 | 2.4 | 2.4 | 0.8 | 1.9 | 0.1 |
| GAD 116-130 | cpm | 759.2 | 361.8 | 2476.3 | 791.6 | 3441.2 | 1973.2 | 717.2 | 1415.3 | 2822.8 | 150.2 | 468.2 | 138.5 | 1225.2 | 414 |
|  | RR | 0.5 | 2.2 | 4.1 | 2.3 | 1.7 | 1.4 | 1.0 | 1.4 | 4.4 | 0.9 | 2.8 | 0.7 | 3.0 | 0.1 |
| GAD 206-220 | cpm | 760.7 | 409.5 | 3675.5 | 257.2 | 8930.8 | 2624.2 | 3600.8 | 1956 | 4217.8 | 270.8 | 779.2 | 143.3 | 1586.8 | 654.5 |
|  | RR | 0.5 | 2.4 | 6.1 | 0.7 | 4.3 | 1.8 | 4.8 | 1.9 | 6.6 | 1.6 | 4.6 | 0.7 | 3.8 | 0.2 |
| GAD 271-285 | cpm | 374.7 | 325.3 | 8206 | 1462.8 | 1312.2 | 2553.6 | 645.2 | 1726.2 | 2029.3 | 170.2 | 136.7 | 152.1 | 1688.5 | 370.7 |
|  | RR | 0.3 | 1.9 | 13.7 | 4.2 | 0.6 | 1.8 | 0.9 | 1.7 | 3.2 | 1.0 | 0.8 | 0.8 | 4.1 | 0.1 |
| GAD 356-370 | cpm | 888.5 | 320 | 3256.3 | 361.8 | 3822.3 | 2240.4 | 863.2 | 1262.5 | 790.2 | 188.2 | 153.2 | 180.4 | 2684.5 | 275 |
|  | RR | 0.6 | 1.9 | 5.4 | 1.0 | 1.8 | 1.6 | 1.1 | 1.2 | 1.2 | 1.1 | 0.9 | 0.9 | 6.5 | 0.1 |
| GAD 376-390 | cpm | 1222.3 | 217.7 | 2573.8 | 226 | 1293 | 2558.2 | 480 | 975.7 | 1622.7 | 144 | 126.2 | 160.5 | 1047 | 237.5 |
|  | RR | 0.8 | 1.3 | 4.3 | 0.7 | 0.6 | 1.8 | 0.6 | 1.0 | 2.5 | 0.9 | 0.8 | 0.8 | 2.5 | 0.1 |
| GAD 431-450 | cpm | 1560.3 | 2502.7 | 2559.3 | 134.4 | 891.2 | 2734.8 | 483 | 1250.8 | 2309.7 | 228 | 238.3 | 128.2 | 4295.3 | 203.3 |
|  | RR | 1.1 | 14.9 | 4.3 | 0.4 | 0.4 | 1.9 | 0.6 | 1.2 | 3.6 | 1.3 | 1.4 | 0.6 | 10.4 | 0.1 |
| GAD 481-495 | cpm | 1405.7 | 482.2 | 2002.7 | 2086.8 | 3356.3 | 3121.2 | 890.2 | 1082.8 | 2837 | 164.7 | 116.8 | 175.3 | 989.5 | 102.2 |
|  | RR | 1.0 | 2.9 | 3.3 | 6.0 | 1.6 | 2.2 | 1.2 | 1.1 | 4.4 | 1.0 | 0.7 | 0.9 | 2.4 | 0.0 |
| GAD 511-525 | cpm | 738.8 | 259.3 | 2315.8 | 259.6 | 1860.8 | 2819.2 | 526.3 | 1257.5 | 1891.2 | 167.7 | 214.5 | 165.5 | 449.2 | 186.2 |
|  | RR | 0.5 | 1.5 | 3.9 | 0.8 | 0.9 | 2.0 | 0.7 | 1.2 | 2.96 | 1.0 | 1.3 | 0.8 | 1.1 | 0.0 |
| GAD 526-540 | cpm | 804.8 | 426.5 | 2397.7 | 576.4 | 1465.2 | 2536 | 436.7 | 778.2 | 441.3 | 361.3 | 219.7 | 151.7 | 861.5 | 529.8 |
|  | RR | 0.6 | 2.5 | 4.0 | 1.7 | 0.7 | 1.8 | 0.6 | 0.8 | 0.7 | 2.1 | 1.3 | 0.8 | 2.1 | 0.1 |
| GAD 536-550 | cpm | 947.8 | 256 | 4372.2 | 236.3 | 2511 | 1809.8 | 711.2 | 1169.5 | 3677.3 | 163.3 | 196.7 | 128.6 | 978 | 541.8 |
|  | RR | 0.7 | 1.5 | 7.3 | 0.7 | 1.2 | 1.3 | 0.9 | 1.1 | 5.8 | 1.0 | 1.2 | 0.6 | 2.4 | 0.1 |
| GAD 546-560 | cpm | 771.2 | 358.5 | 2216.5 | 761.8 | 1917.3 | 2325.4 | 526.8 | 850 | 1737.3 | 231.8 | 242.2 | 155.6 | 663 | 151.2 |
|  | RR | 0.5 | 2.1 | 3.7 | 2.2 | 0.9 | 1.6 | 0.7 | 0.8 | 2.7 | 1.4 | 1.4 | 0.8 | 1.6 | 0.0 |
| GAD 551-565 | cpm | 491.5 | 1389 | 4156 | 338 | 5389 | 3975.4 | 1403.8 | 1527.5 | 2728.5 | 196.8 | 425.7 | 156.9 | 1609.7 | 411.3 |
|  | RR | 0.3 | 8.3 | 6.9 | 1.0 | 2.6 | 2.8 | 1.9 | 1.5 | 4.3 | 1.2 | 2.5 | 0.8 | 3.9 | 0.1 |
| GAD 556-570 | cpm | 1283 | 240.8 | 1460.5 | 1428.4 | 2172.5 | 2745.2 | 490 | 1116.7 | 6097.2 | 186 | 284.7 | 151.2 | 1155.2 | 194.2 |
|  | RR | 0.9 | 1.4 | 2.4 | 4.1 | 1.0 | 1.9 | 0.6 | 1.1 | 9.5 | 1.1 | 1.7 | 0.8 | 2.8 | 0.0 |
| No Ag | cpm | 1448 | 168.2 | 600.1 | 345.6 | 2077.1 | 1434.4 | 754.5 | 1025.1 | 639.3 | 169.3 | 167.7 | 200.4 | 413.3 | 4053.2 |

**Supplementary Table 3.** Proliferative responses (cpm) and relative ratios (RR) against GAD65 peptides compared to absence of peptides stimulation (No Ag) in 14 T1D Sardinian patients. The thick vertical line separates 11 DR4 positive from 3 DR4 negative patients. Patients are identified using a numerical ID; below each ID, the proliferative responses and RR are shown. Patients 01 and 15 were not available for cpm analysis. All values are derived from sextuplet cultures. Shaded areas represent responses that were considered positive (RR≥3) and used in the statistical analysis.

|  | | HLA-DR4/DR4 or HLA-DR4/X | | | | | | | | | | | | | | HLA-DR3/DR3 or HLA-DR3/X | | | |
| --- | --- | --- | --- | --- | --- | --- | --- | --- | --- | --- | --- | --- | --- | --- | --- | --- | --- | --- | --- |
| ID 🡪 | | 01 | 02 | 03 | 04 | 05 | 06 | 07 | 08 | 09 | 10 | 11 | 12 | 13 | 14 | 15 | 16 | 17 | 18 |
| GAD 76-90 | cpm | 431.7 | 7532.8 | 5258.5 | 2550.7 | 462.2 | 4023.3 | 5477.3 | 2129.8 | 1222.8 | 6958.7 | 189 | 490.8 | 1407.2 | 5311.5 | 499.8 | 268 | 326.8 | 1323.2 |
|  | RR | 0.3 | 1.5 | 1.3 | 0.6 | 0.9 | 2.2 | 1.8 | 2.0 | 0.3 | 1.4 | 1.0 | 1.2 | 1.7 | 2.2 | 0.5 | 0.9 | 0.8 | 1.1 |
| GAD 81-95 | cpm | 600.2 | 5246.8 | 2774 | 3878.3 | 694.3 | 980.8 | 2519.7 | 1797.5 | 656 | 8234.2 | 239.3 | 507.7 | 629.8 | 11809.2 | 739.7 | 677.5 | 559 | 1421.2 |
|  | RR | 0.5 | 1.1 | 0.7 | 0.9 | 1.4 | 0.5 | 0.8 | 1.7 | 0.2 | 1.6 | 1.3 | 1.2 | 0.8 | 4.9 | 0.7 | 2.3 | 1.4 | 1.2 |
| GAD 101-115 | cpm | 642 | 8723 | 3456.8 | 5272.2 | 479.8 | 1917.3 | 3673.8 | 2823.7 | 1220.7 | 7089.5 | 197.2 | 489.8 | 892 | 4535.2 | 901.3 | 303.8 | 394.8 | 1404.5 |
|  | RR | 0.5 | 1.8 | 0.9 | 1.2 | 1.0 | 1.0 | 1.2 | 2.7 | 0.3 | 1.4 | 1.1 | 1.2 | 1.1 | 1.9 | 0.9 | 1.1 | 1.0 | 1.2 |
| GAD 116-130 | cpm | 759.5 | 5173 | 1694.7 | 2988 | 663 | 1357 | 1675.3 | 1786.8 | 896 | 3215.3 | 197.7 | 473.3 | 838.2 | 2106.7 | 619.5 | 453.2 | 843 | 1682.7 |
|  | RR | 0.6 | 1.1 | 0.4 | 0.7 | 1.3 | 0.7 | 0.5 | 1.7 | 0.2 | 0.6 | 1.1 | 1.1 | 1.0 | 0.9 | 0.6 | 1.6 | 2.1 | 1.4 |
| GAD 206-220 | cpm | 1382.8 | 25675.2 | 5582.5 | 8237.5 | 1610.5 | 3050.5 | 3771.2 | 6251 | 4165 | 10055.3 | 175.5 | 951.3 | 1306 | 20642 | 3343.2 | 391.5 | 839 | 2541.2 |
|  | RR | 1.1 | 5.2 | 1.4 | 2.0 | 3.2 | 1.7 | 1.2 | 5.9 | 1.1 | 2.0 | 0.9 | 2.3 | 1.6 | 8.5 | 3.3 | 1.4 | 2.1 | 2.1 |
| GAD 271-285 | cpm | 1512.5 | 3869 | 2600 | 5158.2 | 653.3 | 1050.2 | 1950.2 | 2771.8 | 3373.3 | 1635.8 | 365 | 509.2 | 606 | 2405.2 | 767 | 328.8 | 463.7 | 2247 |
|  | RR | 1.2 | 0.8 | 0.7 | 1.2 | 1.3 | 0.6 | 0.6 | 2.6 | 0.9 | 0.3 | 1.9 | 1.2 | 0.7 | 1.0 | 0.8 | 1.1 | 1.2 | 1.9 |
| GAD 356-370 | cpm | 1528.7 | 2784.7 | 2684 | 992.5 | 1696.8 | 1755.8 | 3670.3 | 1221 | 1143.5 | 1030.5 | 332.2 | 541.5 | 2086.8 | 2639.3 | 460.3 | 493.8 | 507.2 | 1948.7 |
|  | RR | 1.2 | 0.6 | 0.7 | 0.2 | 3.4 | 1.0 | 1.2 | 1.2 | 0.3 | 0.2 | 1.8 | 1.3 | 2.5 | 1.1 | 0.5 | 1.7 | 1.3 | 1.6 |
| GAD 376-390 | cpm | 514.2 | 3170 | 4423 | 3045 | 600.3 | 2621.2 | 3726.5 | 1670 | 973.7 | 3945.3 | 182.2 | 340.5 | 797.5 | 1919.7 | 1905.5 | 344.8 | 417.8 | 1139.8 |
|  | RR | 0.4 | 0.6 | 1.1 | 0.7 | 1.2 | 1.4 | 1.2 | 1.6 | 0.3 | 0.8 | 1.0 | 0.8 | 1.0 | 0.8 | 1.9 | 1.2 | 1.1 | 1.0 |
| GAD 431-450 | cpm | 403 | 6388.7 | 2349.8 | 3482 | 279 | 2983.5 | 5694.7 | 2178.3 | 498 | 8593.8 | 354.2 | 315.8 | 608.8 | 2306.5 | 596.3 | 201.8 | 441 | 1383.5 |
|  | RR | 0.3 | 1.3 | 0.6 | 0.8 | 0.6 | 1.6 | 1.8 | 2.1 | 0.1 | 1.7 | 1.9 | 0.8 | 0.7 | 1.0 | 0.6 | 0.7 | 1.1 | 1.2 |
| GAD 481-495 | cpm | 326.3 | 1391.3 | 1695.2 | 1463.7 | 1019.3 | 2515.7 | 2960.7 | 766.3 | 2117 | 2099.2 | 180.2 | 426.5 | 998.7 | 1011 | 1485 | 194.5 | 373.5 | 1607.7 |
|  | RR | 0.3 | 0.3 | 0.4 | 0.3 | 2.1 | 1.4 | 1.0 | 0.7 | 0.6 | 0.4 | 1.0 | 1.0 | 1.2 | 0.4 | 1.5 | 0.7 | 1.0 | 1.3 |
| GAD 511-525 | cpm | 443 | 6272.7 | 3847.3 | 2582 | 571.5 | 2749.8 | 5301.8 | 1652.5 | 1453.5 | 6195.4 | 204.2 | 359.7 | 464 | 4286 | 1753 | 220.5 | 330.2 | 1382.2 |
|  | RR | 0.3 | 1.3 | 1.0 | 0.6 | 1.2 | 1.5 | 1.7 | 1.6 | 0.4 | 1.2 | 1.1 | 0.9 | 0.6 | 1.8 | 1.7 | 0.8 | 0.8 | 1.2 |
| GAD 526-540 | cpm | 376 | 7154.3 | 4205.8 | 3230 | 503.2 | 4454 | 5357.2 | 2207 | 765.5 | 4700.5 | 328.2 | 351.7 | 466.8 | 4258.3 | 1216.5 | 484.5 | 190.2 | 1203.3 |
|  | RR | 0.3 | 1.5 | 1.1 | 0.8 | 1.0 | 2.4 | 1.7 | 2.1 | 0.2 | 0.9 | 1.7 | 0.8 | 0.6 | 1.8 | 1.2 | 1.7 | 0.5 | 1.0 |
| GAD 536-550 | cpm | 1452.3 | 7712.2 | 2048.3 | 5417.2 | 472 | 2382.2 | 3113.7 | 2684.2 | 967 | 8420.8 | 180.8 | 533.7 | 774 | 4835.3 | 349.3 | 570.5 | 439.2 | 1055.5 |
|  | RR | 1.1 | 1.6 | 0.5 | 1.3 | 1.0 | 1.3 | 1.0 | 2.5 | 0.3 | 1.7 | 1.0 | 1.3 | 0.9 | 2.0 | 0.3 | 2.0 | 1.1 | 0.9 |
| GAD 546-560 | cpm | 249 | 6308 | 4078 | 3257.2 | 524.8 | 1941.3 | 6250.5 | 1903.8 | 895 | 7325.7 | 196.5 | 498 | 597.5 | 4619.7 | 808 | 287.2 | 281.8 | 1199.5 |
|  | RR | 0.2 | 1.3 | 1.0 | 0.8 | 1.1 | 1.1 | 2.0 | 1.8 | 0.2 | 1.5 | 1.0 | 1.2 | 0.7 | 1.9 | 0.8 | 1.0 | 0.7 | 1.0 |
| GAD 551-565 | cpm | 1067.2 | 14606.5 | 5968.5 | 5757.5 | 1385 | 4544.7 | 5466.7 | 2803.2 | 1917.7 | 11610.8 | 149.5 | 384.7 | 1221.7 | 12173 | 1521.8 | 810.2 | 794 | 1788 |
|  | RR | 0.8 | 2.98 | 1.5 | 1.4 | 2.8 | 2.5 | 1.8 | 2.7 | 0.5 | 2.3 | 0.8 | 0.9 | 1.5 | 5.0 | 1.5 | 2.8 | 2.0 | 1.5 |
| GAD 556-570 | cpm | 1053.8 | 13430.8 | 8064 | 4575.7 | 749 | 4823.8 | 4681.8 | 3249.3 | 976 | 6185.8 | 190.3 | 364 | 1338.3 | 9415.7 | 1135.2 | 230.5 | 327.8 | 1290.5 |
|  | RR | 0.8 | 2.7 | 2.0 | 1.1 | 1.5 | 2.6 | 1.5 | 3.1 | 0.3 | 1.2 | 1.0 | 0.9 | 1.6 | 3.9 | 1.1 | 0.8 | 0.8 | 1.1 |
| No Ag | cpm | 1278.5 | 4909.6 | 3952.2 | 4222.2 | 496.8 | 1836.7 | 3086.3 | 1053.4 | 3817.8 | 5051.5 | 187.8 | 415.8 | 834.2 | 2423.8 | 1007.8 | 288.8 | 393.1 | 1199.5 |

**Supplementary Table 4.** Proliferative responses (cpm) and relative ratios (RR) against GAD65 peptides compared to absence of peptides stimulation (No Ag) in 18 healthy controls. The thick vertical line separates 14 DR4 positive from 4 DR4 negative individuals. Control individuals are identified using a numerical ID; below each ID the cpm and RR are shown. All values are derived from sextuplet cultures. Shaded areas represent responses that were considered positive (RR≥3) and used in the statistical analysis.
